# Radiographic findings of Desbuquois dysplasia

**DOI:** 10.1259/bjrcr.20200137

**Published:** 2020-11-02

**Authors:** Meltem Özdemir, Rasime Pelin Kavak

**Affiliations:** 1 Department of Radiology, University of Health Sciences Dışkapı Yıldırım Beyazıt Training and Research Hospital, Ankara, Turkey

## Abstract

Desbuquois dysplasia is an autosomal recessive chondrodysplasia characterized by severe micromelic dwarfism, joint laxity, progressive scoliosis, and advanced carpotarsal ossification. Two different types of Desbuquois dysplasia have been identified according to the presence (Type 1) or absence (Type 2) of characteristic hand abnormalities including bifid distal thumb phalanx, an extra ossification center distal to the second metacarpal, and interphalangeal joint dislocations. Further, Kim et al have described a milder variant of Desbuquois dysplasia characterized by short stature and hands with short metacarpals, elongated proximal and distal phalanges, and extremely advanced carpal ossification. Here, we present a 19-year-old male patient with Kim variant of Desbuquois dysplasia. He displays almost all of the characteristic skeletal findings of Desbuquois dysplasia along with the characteristic hand features described by Kim et al. This patient is unique in that he also presents sagittal femoral bowing, a radiographic finding that accompanies various skeletal dysplasias but has never been reported in a patient with Desbuquois dysplasia to date.

## Case presentation

A 19-year-old young male with short stature admitted to the hospital for compulsory health screening to qualify for disability benefits from the state social insurance agency. He was a university student who complained of falling for no reason at unexpected times. He reported that he had been seriously injured in recent years due to these unexpected falls and his arm was broken in the previous year. So, he started going to school every day under the supervision of his mother. He is the second child born to non-consanguineous healthy parents of Turkish descent. His elder sister is healthy. There is no individual with short stature or any kind of congenital skeletal abnormalities in the extended family. Our patient was uneventfully born by vaginal delivery at full term. He was born as a small baby and has always been shorter than his peers throughout his entire growth process. He walked late compared to his peers and since he fell frequently, he was exempted from sports classes throughout his education life. However, his intelligence development and school success were sufficient. He finished high school at the same time as his peers. No other significant health problem is present in the history of his childhood. 2 years ago, a molecular analysis of the whole family was carried out in another university hospital. Sanger sequencing showed a homozygous mutation in the patient’s CANT1 gene [c.375 *G* < C (p. Trp125Cys)] and the same type of heterozygous mutation in both parent’s CANT1 genes.

**Figure 1.**
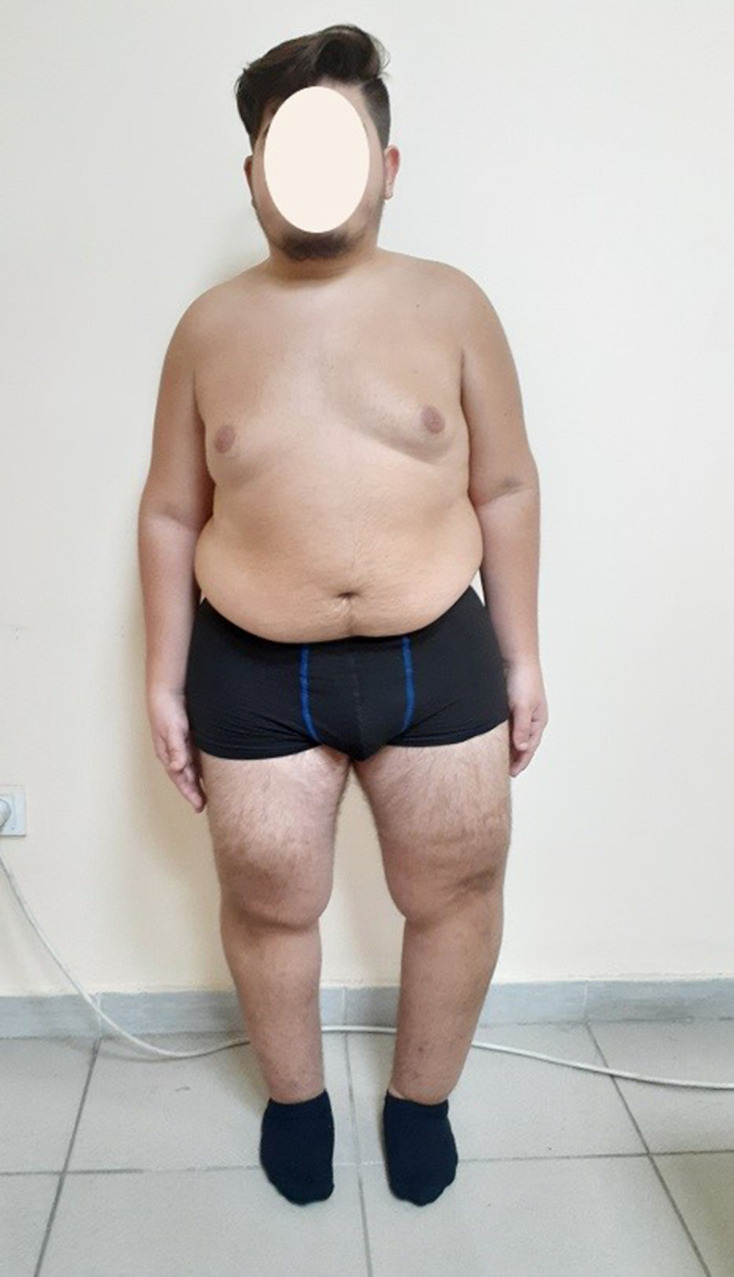
Clinical photograph of the 19-year-old patient with Desbuquois dysplasia.

**Figure 2.**
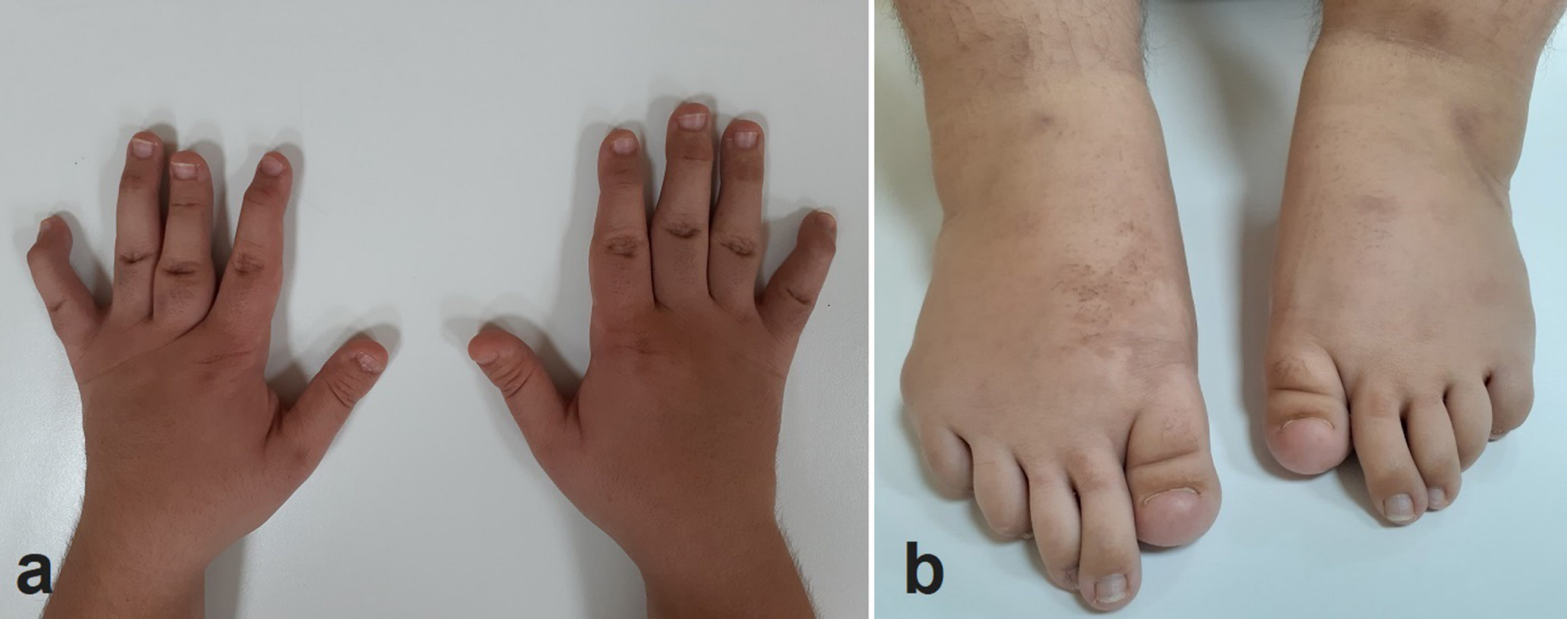
Clinical photographs of the patient’s hands (a) and feet (b). There are flexion contractures in the distal interphalangeal joints of second to fifth fingers and ulnar deviation of the fifth fingers of both hands. The fourth finger of the left hand is longer than the second and the third (a). Both big toes are abnormally short and both of their nails are dysplastic. The second and third toes are elongated and flexed (b).

He was 148 cm tall (−3.5 SD) and weighed 70 kg (body mass index: 32, within the limits of obesity). He had disproportionately short limbs, consistent with micromelic dwarfism. No facial dysmorphic features were noted except for mild mid-face hypoplasia. He had bilateral genu varum, patellar dislocation, and hyperextensibility of the knees (Figure 1). His hands appeared abnormal with flexion contracture in the distal interphalangeal joints of second to fifth fingers (except in that of the third finger of the left hand) and ulnar deviation of the fifth fingers. The fourth finger of the left hand was longer than the second and the third. Both big toes were abnormally short and both of their nails were dysplastic. The second and third toes were elongated and flexed (Figure 2).

## Imaging findings

**Figure 3.**
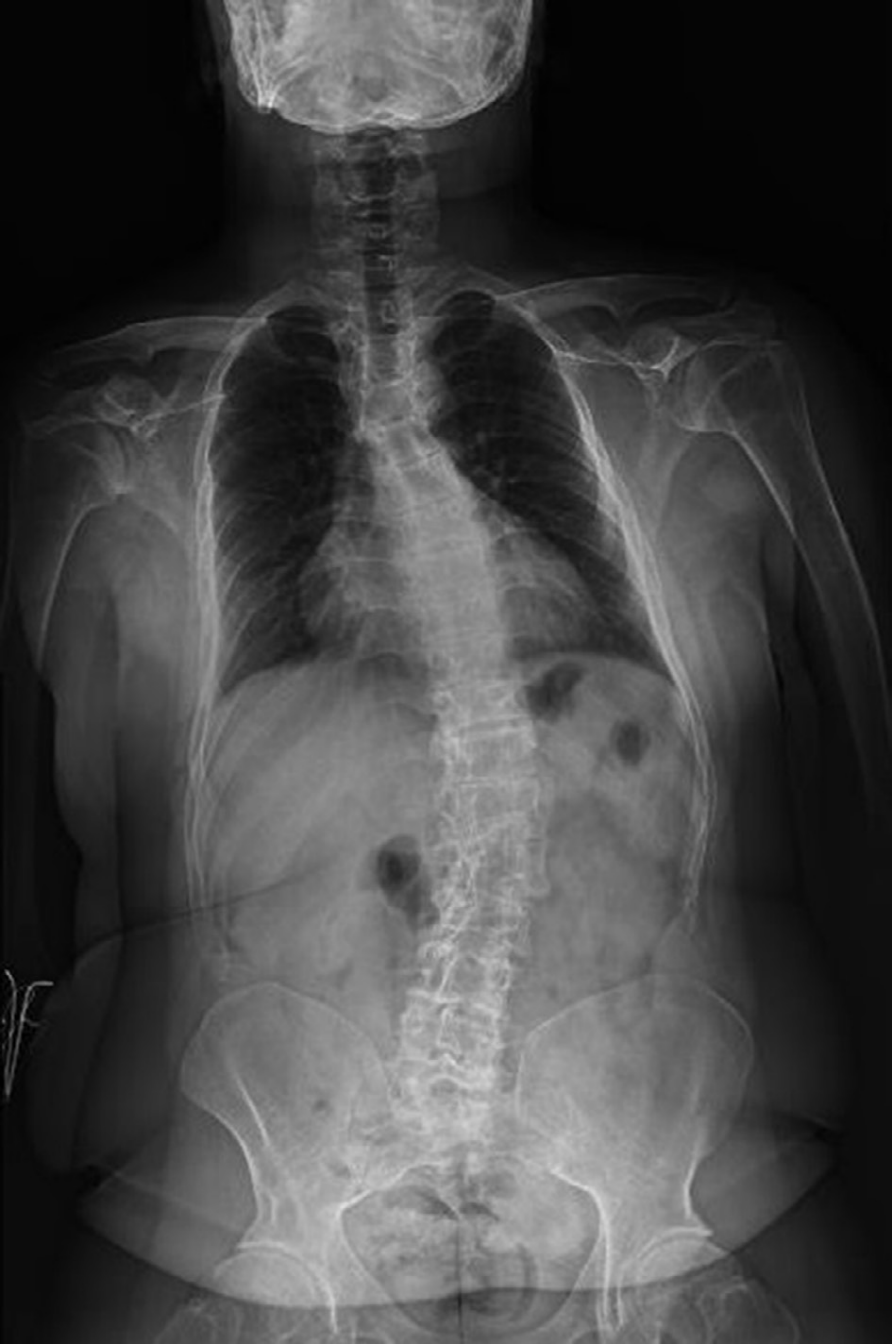
Anteroposterior full-length radiographic view of the spine. There is S-shape scoliosis with the upper curve convex to the right and the lower curve convex to the left. There are also vertebral end-plate irregularities and narrowed disc spaces as evidence of degenerative spondylosis. The chest is narrow with an increased cardiothoracic ratio.

**Figure 4.**
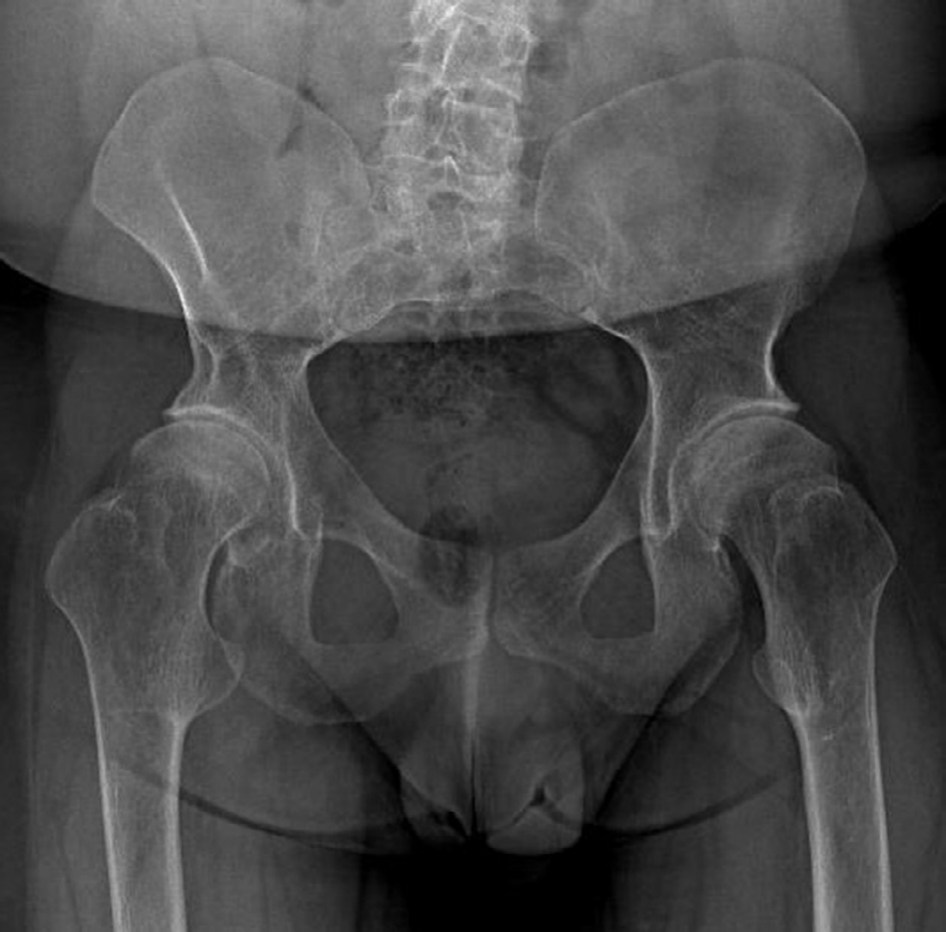
Antero posterior pelvis radiograph shows short and wide femoral necks with enlarged and elevated greater trochanters. Lesser trochanters, as well as intertrochanteric region, are prominent.

**Figure 5.**
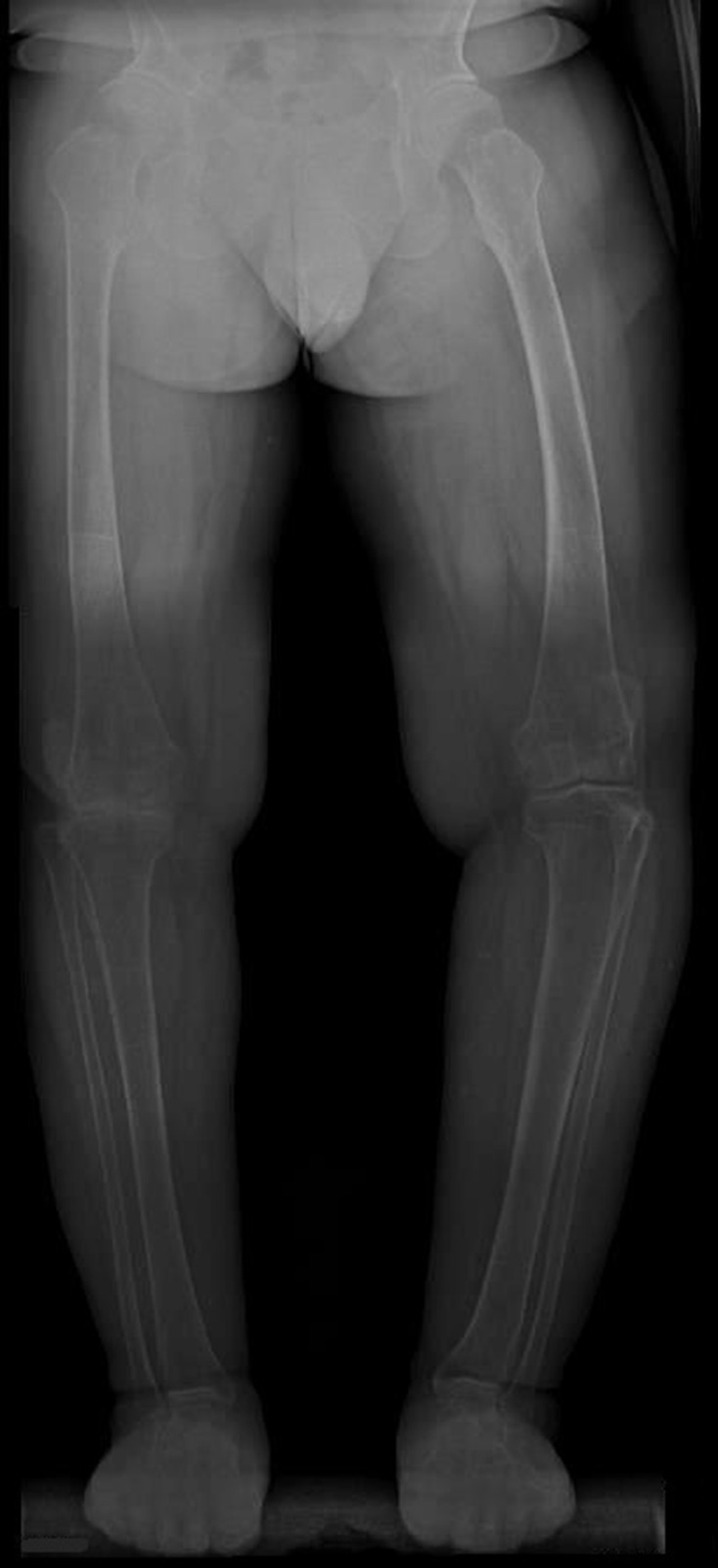
Full-length standing anteroposterior radiograph of both lower extremities shows genu varum deformity and precocious osteoarthritic changes in both hip, knee and ankle joints.

**Figure 6.**
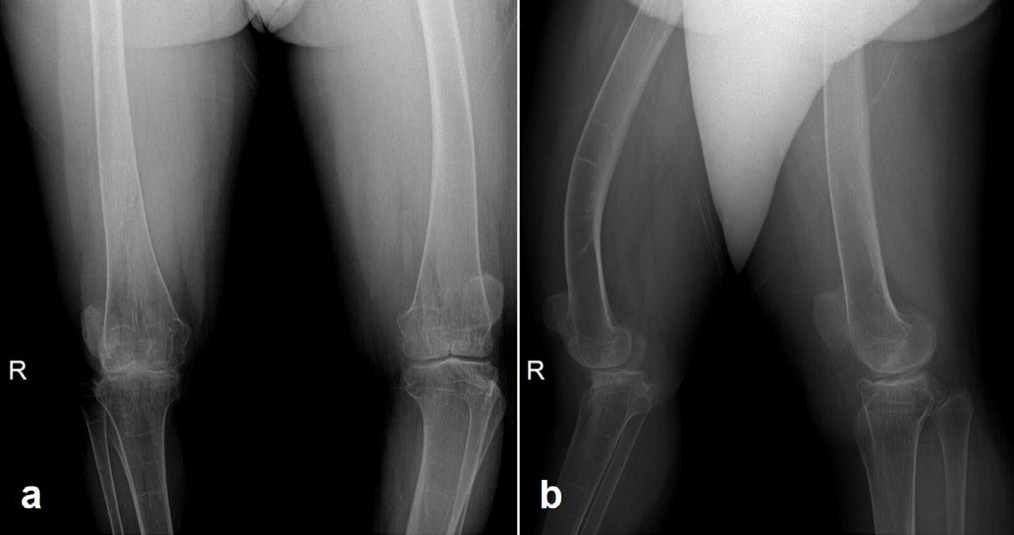
Anteroposterior (a) and lateral (b) knee radiographs demonstrate bilateral knee osteoarthritis along with anterior-bowing of the femoral shafts, both of which were more pronounced on the right side. Metaphyses of both femora and tibiae are enlarged and joint surfaces are flattened. There is bilateral patellar dislocation.

**Figure 7.**
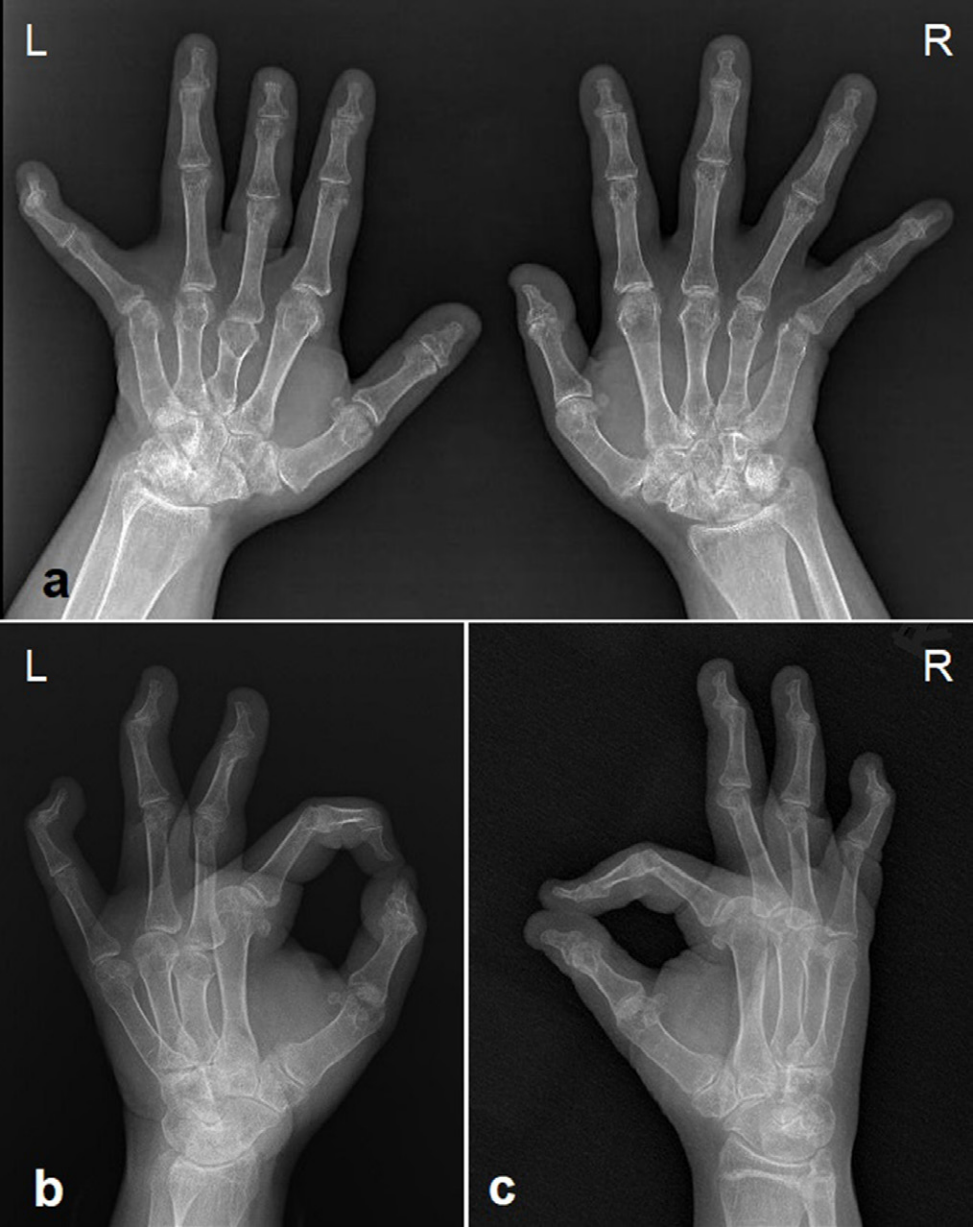
Antero posterior (a) and lateral (b, c) hand radiographs show short metacarpals (more pronounced in the third metacarpal of the left hand) and slightly elongated proximal and middle phalanges. Distal phalanges are short. And fixed flexion of the distal interphalangeal joints of the second to fifth fingers of both hands (except the third finger of the left hand) is evident. Bilateral carpal coalition secondary to osteoarthritis is remarkable.

**Figure 8.**
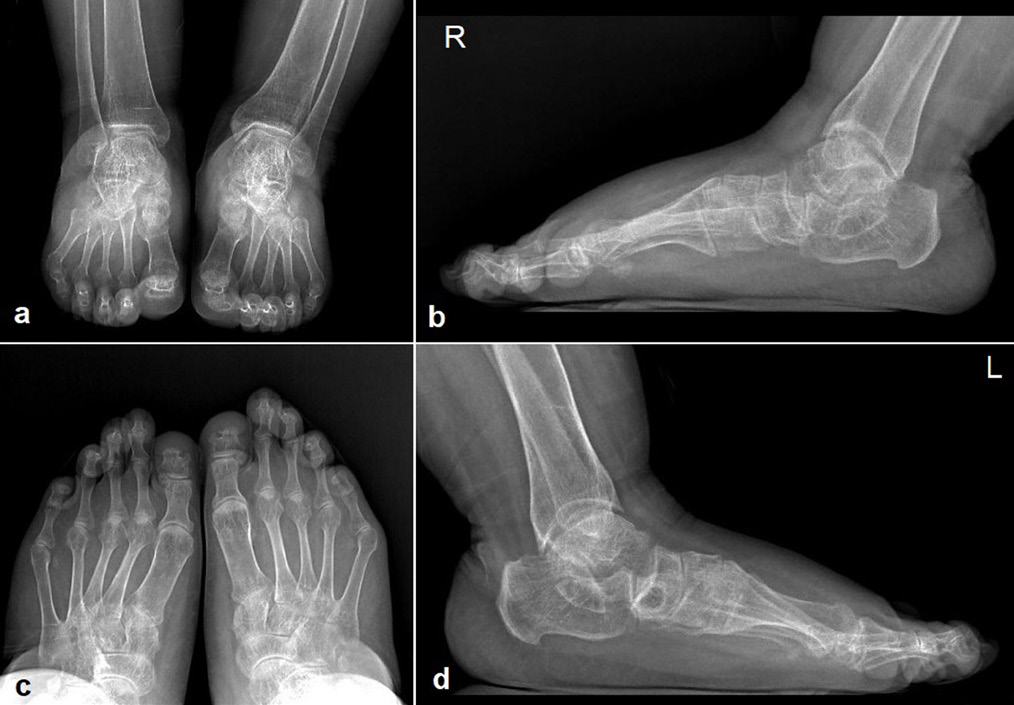
Anteroposterior ankle (a), lateral right foot and ankle (b), anteroposterior foot (c), and lateral left foot and ankle (b) radiographs demonstrate narrow joint space and periarticular sclerosis in almost all of the included joints, as the result of early osteoarthritis. The phalanges and metatarsals of the big toes are short and wide, and the phalanges of the second and third toes are elongated (c). There are plantar flexion of calcanea and decreased calcaneal inclination angles (b, d).

The radiographic survey revealed generalized osteopenia. The dual-energy X-ray absorptiometry (DXA) scan revealed a T-score of −3.9. On the anteroposterior (AP) full-length radiographic view of the spine, there was S-shape scoliosis with the upper curve convex to the right and the lower curve convex to the left. Vertebral end-plate irregularities and narrowed disc spaces, as evidence of degenerative spondylosis, were present. The chest was narrow with an increased cardiothoracic ratio (Figure 3). AP pelvis radiograph showed that the femoral necks were short and wide. Besides, both greater trochanters were enlarged and elevated. Lesser trochanters, as well as intertrochanteric regions, were prominent (Figure 4). Full-length standing AP radiograph of both lower extremities showed genu varum deformity. Precocious osteoarthritic changes were evident in both hip, knee and ankle joints, more prominent on the right side (Figure 5). AP and lateral (L) knee radiographs demonstrated bilateral anterior-bowing of the femoral shafts and knee osteoarthritis, both of which were more pronounced on the right side. Metaphyses of both femora and tibiae were enlarged and joint surfaces were flattened. There was bilateral patellar dislocation (Figure 6). Short metacarpals (more pronounced in the third metacarpal of the left hand) and slightly elongated proximal and middle phalanges were observed on AP and L radiographs of both hands. Distal phalanges were short. And fixed flexion of the distal interphalangeal joints of the second to fifth fingers of both hands (except the third finger of the left hand) was noted. The bilateral carpal coalition secondary to osteoarthritis was remarkable (Figure 7). Biplanar foot and ankle radiographs demonstrated narrow joint spaces and periarticular sclerosis in almost all of the included joints, as the result of early osteoarthritis. The metatarsals, as well as the phalanges, of the big toes, were short and wide. But the phalanges of the second and third toes were elongated. There were plantarflexion of calcanea and decreased calcaneal inclination angles (Figure 8).

In comprehensive clinical and radiological examinations, no cardiopulmonary disorders, including cardiac septal defect and respiratory distress, were detected. And the ophthalmologic examination of the patient revealed no glaucoma. Based on the rather specific molecular and radiographic findings, the patient was diagnosed as Kim variant of DBQD.

## Treatment

A regular physical therapy program was planned for early-onset osteoarthritis. The patient was educated about the course and prognosis of the disease. Genetic counseling was provided to the patient and other family members.

## Discussion

Desbuquois dysplasia (DBQD), an autosomal recessive chondrodysplasia characterized by severe micromelic dwarfism, joint laxity, progressive scoliosis, and advanced carpotarsal ossification, is an extremely rare disorder with fewer than 50 cases reported to date. Two different types of DBQD have been identified depending on whether or not the following specific hand abnormalities are present in the phenotype: bifid distal thumb phalanx, an extra ossification center distal to the second metacarpal, and dislocation of the interphalangeal joints. Cases with these additional hand anomalies are classified as Type 1, and those without them are classified as Type 2. Further, Kim et al have described a milder variant of DBQD characterized by short stature and hands with short metacarpals, elongated proximal and distal phalanges, and extremely advanced carpal ossification. Both the Types 1 and 2 and the Kim variant have been shown to result from mutations in the calcium-activated nucleotidase 1 (CANT1) gene [DBQD-1 (MIM 251450)].^1^ Besides, cases of DBQD showing mutations in the xylosyltransferase 1 (XYLT1) gene have also been reported [DBQD-2 (MIM 615777)].^2^

DBQD typically presents with severe pre- and post-natal growth retardation and exhibits characteristic phenotypic and radiographic features from the pre-natal period. However, the disease shows a significant heterogeneity in terms of clinical course. Furthermore, the characteristic phenotypic and radiographic findings evolve as the child grows, making diagnosis more difficult in older children and adults compared to that in young children.^3,4^ In the current literature, there is a paucity of data on the radiographic appearance of DBQD in adults. Here, we presented a young male with Kim variant of DBQD, with emphasis on radiographic features.

According to the previously reported cases, DBQD is mostly manifested by severe growth retardation and characteristic findings that are evident from the pre-natal period. Facial dysmorphic features include a flat face with prominent eyes and hypoplastic midface, micrognathia, and a long upper lip with flat philtrum. The distinctive radiographic features of the disorder are short long bones showing metaphyseal splay, an abnormal trochanteric structure so-called 'Swedish key' appearance, and advanced carpal and tarsal bone age. Various clinical features including, mental retardation, glaucoma, lung hypoplasia, cardiac septal defects, and obesity may accompany skeletal abnormalities.^4,5^ DBQD offers a significant heterogeneity in terms of clinical course ranging from fetal hydrops to an almost healthy life reaching to adulthood, except for various difficulties with the skeletal system.^6,7^ Although he showed almost all of the characteristic skeletal findings of DBQD, the patient we presented was a relatively lucky one who displayed a clinical picture that is closer to the healthy side of the clinical spectrum. He did not have severe lung hypoplasia which has been reported to be responsible for most deaths in early childhood among patients with DBQD. His intelligence level was not below his peers, and he had no facial dysmorphic features except for mild midfacial hypoplasia. Further, while growth has been reported being extremely lagging with standard deviation scores between −4 and −10 in most DBQD cases, our patient’s growth retardation was relatively mild with a standard deviation score of −3.5. On the other hand, his skeletal findings were rich and not mild at all.

Our patient presented with micromelic dwarfism, and ambulatory difficulties due to significant joint laxity. Besides, he had generalized osteopenia, scoliosis, thoracic hypoplasia, genu varum, and precocious osteoarthritic changes, all of which are among the main radiographic features attributed to DBQD. He had short and enlarged femoral necks with prominent lesser trochanters. However, he did not show the characteristic ‘Swedish key’ appearance, a distinctive radiographic feature of DBQD, which is characterized by the enlargement of the lesser trochanters with metaphyseal beaking.^6^ Likewise, vertebral coronal clefting, another common radiographic finding observed in children with DBQD, was not present in our case.^8^ Faivre et al pointed out that the Swedish key appearance of the proximal femora becomes less pronounced and the vertebral clefts disappear with age.^4^ Supporting the observation of Faivre et al, none of the four adult patients (between the ages of 16 and 22) presented by Kim et al showed the characteristic Swedish key appearance or vertebral coronal clefting.^9^

DBQD offers remarkably distinctive hand abnormalities including bifid distal thumb phalanx, an extra ossification center distal to the second metacarpal, and dislocation of the interphalangeal joints. However, these highly specific hand abnormalities have been reported to exist in only one-third of all cases.^6^ Further, Nizon et al have shown that also another combination of hand abnormalities, including multiple finger dislocations, interphalangeal epiphyseal anomalies, and thumb digitization, without any extra ossification center, can be observed in patients with CANT1 mutations.^10^ Our patient showed none of these mentioned features. The phenotypic, as well as radiographic features of his hands, were very similar to those of patients reported by Kim et al.^9^ Both our patient and those of Kim et al showed short metacarpals, slightly elongated proximal and middle phalanges, short distal phalanges, almost equal length of second to fourth fingers, fixed flexion of the distal interphalangeal joints, ulnar deviation of the fifth finger, and severe carpal coalition. Besides, the phenotypic and radiographic features of our patient’s feet were also very similar with those of Kim et al.: short and wide metatarsals and phalanges of the great toes, and elongated and flexed second and third toes. On the other hand, the radiographic features of the feet of our patient differed from those of their patients in that he did not show metatarsus adductus or planus valgus.^9,11^ The resemblance between our patient and the patients of Kim et al was not limited only to their hands and feet. They were very similar to each other also in terms of normal intelligence levels, absence of serious clinical findings such as cardiopulmonary disorders, more favorable growth retardation, and radiographic features of the spine and proximal femora. These close similarities strongly suggested the diagnosis of Kim variant of DBQD in our patient.

Our patient demonstrated bilateral anterior-bowing of the femoral shafts. Sagittal bowing is, to a certain extent, a characteristic of the human femur.^12^ However, the inclination in the sagittal or coronal plane that reaches angles beyond normal anatomical boundaries is a pathological finding that accompanies various skeletal dysplasias such as acrofacial dysostosis, Antley-Bixler syndrome, kyphomelic dysplasia, metaphyseal chondrodysplasia, osteogenesis imperfecta, spondyloepimetaphyseal dysplasia, and short-rib thoracic dysplasia.^13^ To date, anterior femoral bowing has not been reported in any DBQD cases, including those with the Kim variant. Since we do not have access to the previous clinical records and radiological images of the patient, we do not have information about whether this finding is congenital or developed in the following years. But in both cases, this additional finding appears to expand the radiographic spectrum of DBQD, including that of the Kim variant. It is clear, however, that much more case reports are needed to make an accurate radiographic description of the disorder.

## Learning points

1. DBQD is an autosomal recessive chondrodysplasia characterized by severe micromelic dwarfism, joint laxity, progressive scoliosis, and advanced carpotarsal ossification.
2. Two different types of DBQD have been identified according to the presence (Type 1) or absence (Type 2) of characteristic hand abnormalities including bifid distal thumb phalanx, an extra ossification center distal to the second metacarpal, and interphalangeal joint dislocations.
3. Kim variant of DBQD is a milder variant of the disease that is characterized by short stature and hands with short metacarpals, elongated proximal and distal phalanges, and extremely advanced carpal ossification.
4. DBQD typically presents with severe pre- and post-natal growth retardation and exhibits characteristic phenotypic and radiographic features from the pre-natal period.
5. Anterior-bowing of the femoral shafts may be noted in adult patients with DBQD.
